# Supplementary material for: Integrated Analysis of the Lung Microbiome and Metabolome Reveals Associations Between Amino Acid Metabolism and Pulmonary Fibrosis in a Bleomycin-Induced Mouse Model
Source: Int J Mol Sci. 2026 Jun 30;27(13):5895. doi: 10.3390/ijms27135895 (PMC13362081; doi:10.3390/ijms27135895)
Supplement: Supplementary file 1 [file ijms-27-05895-s001.zip › result/4.MetDiffAnalysis/B_28d.vs.C_28d/B_28d.vs.C_28d_all_heatmap.pdf]

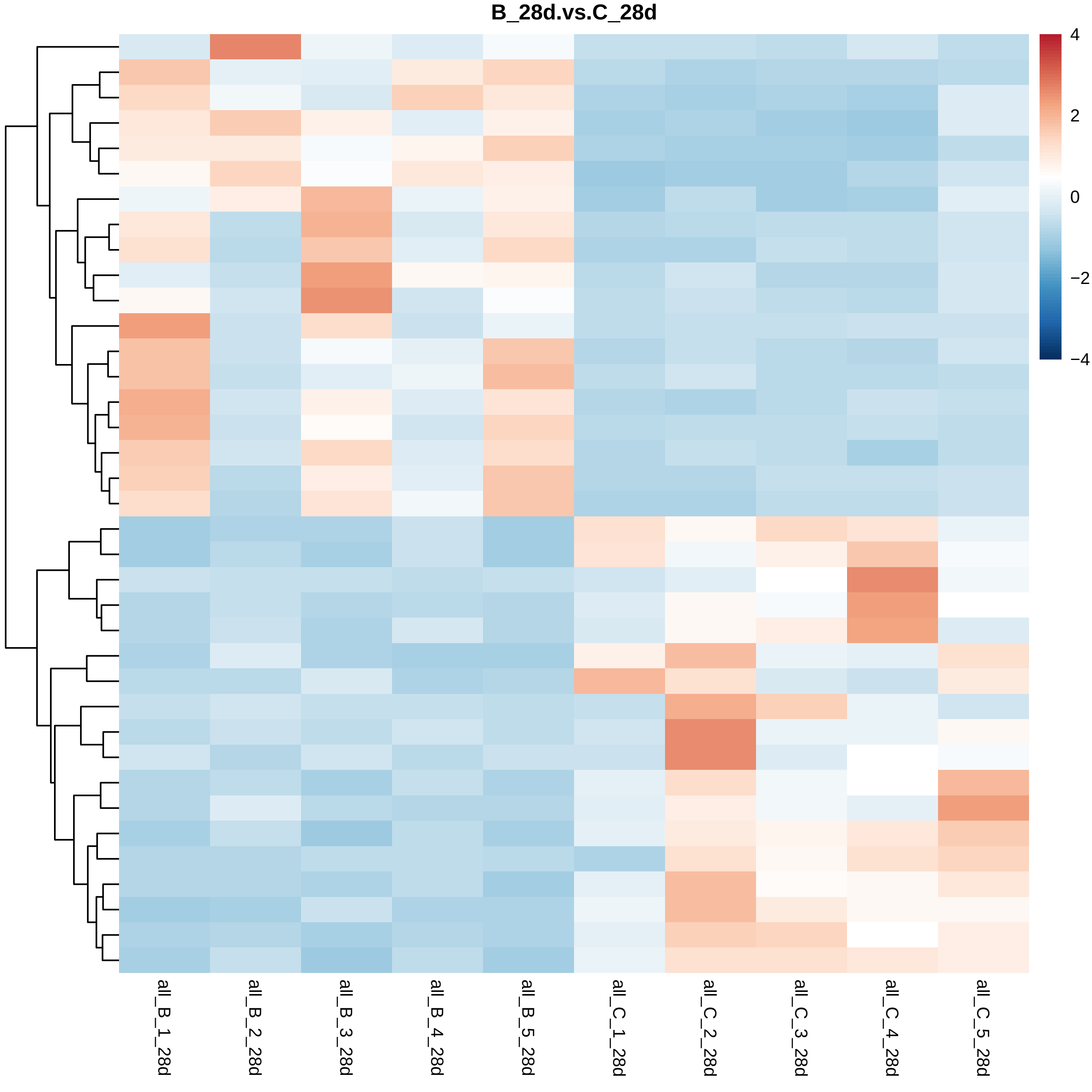

B_28d.vs.C_28d
4
2
0
−2
−4
all_B_1_28d
all_B_2_28d
all_B_3_28d
all_B_4_28d
all_B_5_28d
all_C_1_28d
all_C_2_28d
all_C_3_28d
all_C_4_28d
all_C_5_28d
